# Multivariable Mendelian Randomization: The Use of Pleiotropic Genetic Variants to Estimate Causal Effects

**DOI:** 10.1093/aje/kwu283

**Published:** 2015-01-27

**Authors:** Stephen Burgess, Simon G. Thompson

**Keywords:** causal inference, epidemiologic methods, instrumental variables, lipid fractions, Mendelian randomization, pleiotropy

## Abstract

A conventional Mendelian randomization analysis assesses the causal effect of a risk factor on an outcome by using genetic variants that are solely associated with the risk factor of interest as instrumental variables. However, in some cases, such as the case of triglyceride level as a risk factor for cardiovascular disease, it may be difficult to find a relevant genetic variant that is not also associated with related risk factors, such as other lipid fractions. Such a variant is known as pleiotropic. In this paper, we propose an extension of Mendelian randomization that uses multiple genetic variants associated with several measured risk factors to simultaneously estimate the causal effect of each of the risk factors on the outcome. This “multivariable Mendelian randomization” approach is similar to the simultaneous assessment of several treatments in a factorial randomized trial. In this paper, methods for estimating the causal effects are presented and compared using real and simulated data, and the assumptions necessary for a valid multivariable Mendelian randomization analysis are discussed. Subject to these assumptions, we demonstrate that triglyceride-related pathways have a causal effect on the risk of coronary heart disease independent of the effects of low-density lipoprotein cholesterol and high-density lipoprotein cholesterol.

Mendelian randomization employs genetic variants as instrumental variables to estimate the causal effect of a risk factor on an outcome using observational data, even in the presence of unmeasured confounding (1, 2). A genetic variable is a valid instrumental variable if

- the variant is associated with the risk factor of interest,
- the variant is not associated with any confounder of the risk factor–outcome association, and
- the variant is conditionally independent of the outcome given the risk factor and confounders (3, 4).

These assumptions can be illustrated using a causal directed acyclic graph, displaying a causal effect of one variable on another by an arrow and the absence of a direct causal effect by the lack of an arrow (Figure 1) (5). Although a genetic variant need not be causally associated with the risk factor to be a valid instrumental variable, we assume that there exists a causal variant for which the measured variant is a proxy (6).

**Figure 1.**
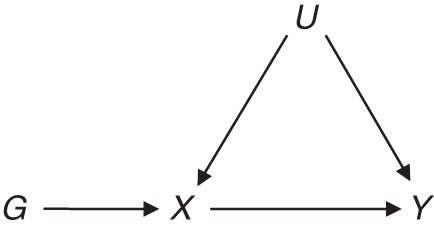
Mendelian randomization assumptions for variant *G* with risk factor *X* in a confounded association with outcome *Y*. Confounders represented by *U* are assumed to be unknown.

In order to avoid violations of the second and third instrumental-variable assumptions, Mendelian randomization experiments have generally relied on genetic variants which are associated with a single risk factor. In practice, however, many variants are pleiotropic—that is, associated with multiple risk factors. Indeed, in some cases, there may be no variants which are solely associated with the risk factor of interest, and a Mendelian randomization analysis cannot be performed without considering pleiotropic variants. In any case, it may desirable to include information on pleiotropic variants in order to provide a more powerful analysis, provided that this does not prejudice its validity. It may also be that multiple quantitative traits relating to the same risk factor are of interest; for example, in cardiovascular disease, the concentration of lipoprotein(a) and the size of lipoprotein(a) particles (7). In this case, the relative proportions of risk reduction associated with interventions separately targeting lipoprotein(a) concentrations and the size of lipoprotein(a) particles may be of interest, and the traits may be regarded as independent risk factors, even if the same genetic variants influence both traits. The possibility of including multiple risk factors in an instrumental-variable analysis is discussed in many econometric textbooks (8), and applied instrumental-variable analyses involving multiple risk factors have been performed (9, 10), but we are unaware of any application of the approach in genetic epidemiology.

The context of this paper is that there are measurements on multiple genetic variants and several associated risk factors, the causal effect of at least 1 of which on the outcome is of interest. We assume that the genetic variants do not influence the outcome via any pathway except those fully mediated by one of the measured risk factors or by some combination of the measured risk factors. Questions about variants with potentially unmeasured or unknown pleiotropic associations are reserved for the Discussion section. We initially discuss how pleiotropic associations may arise and the methods and assumptions necessary for estimating causal effects with several risk factors. We demonstrate the use of these methods in an applied example and then construct a simulation study with parameters chosen to be similar to those in the example to investigate how the methods perform. Finally, we discuss the application of the methods in epidemiologic practice and the interpretation of the applied example.

## METHODS

### Mechanisms for association with multiple risk factors

**Figure 2.**
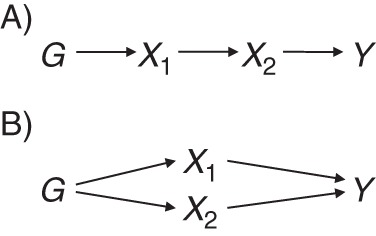
Causal directed acyclic graph illustrating vertical (A) and functional (B) pleiotropy in associations between variant *G*, risk factors *X*_1_ and *X*_2_, and outcome *Y*.

There are several causal mechanisms by which a genetic variant may be associated with multiple risk factors (11). We divide the possible mechanisms into 2 cases (Figure 2): 1) vertical pleiotropy, where a variant is associated with multiple risk factors due to the causal effect of the primary risk factor on a secondary trait, and 2) functional pleiotropy, where the genetic variant is associated with multiple pathways. These 2 cases are not mutually exclusive; it is possible for both of them to exist for the same variant.

In the case of vertical pleiotropy, genetic variants associated with the primary risk factor would be expected to show consistent associations with the secondary trait. For example, genetic variants associated with higher body mass index (weight (kg)/height (m)^2^) would be expected to show a consistent association with higher blood pressure. In this case, the causal effect of body mass index on the outcome would include an indirect effect, mediated through blood pressure, as well as a direct effect comprising all other pathways from body mass index to the outcome that are not operating via blood pressure. If it is assumed that the only pathway by which the genetic variants are associated with the secondary trait is via the primary risk factor, then a simple Mendelian randomization analysis would consistently estimate the causal effect of the primary risk factor on the outcome in spite of the apparent pleiotropic association.

In the case of functional pleiotropy, we suppose there are multiple genetic variants (at least as many variants as there are risk factors) which have different magnitudes of effect on the risk factors. These genetic variants can be used to estimate the causal effects of each risk factor even if none of the variants are specifically associated with any 1 particular risk factor. Since Mendelian randomization is analogous to a randomized trial (12), the use of genetic variants to assess the causal effects of multiple risk factors in a single study is analogous to a factorial randomized trial (see Web Figure 1, available at http://aje.oxfordjournals.org/), where multiple randomized interventions are simultaneously assessed (13). We refer to such an analysis as “multivariable Mendelian randomization.”

### Assumptions

For a multivariable Mendelian randomization analysis to be valid, the genetic variants must satisfy a similar set of assumptions as a conventional instrumental variable, but in this case the variants must be exclusively associated not with a single risk factor but with a set of measured risk factors. It is not necessary for each variant to be associated with every risk factor in the set, but a variant cannot have associations with the outcome except via the risk factors of interest. Specifically, for each variant, we assume that

- the variant is associated with 1 or more of the risk factors,
- the variant is not associated with a confounder of any of the risk factor–outcome associations, and
- the variant is conditionally independent of the outcome given the risk factors and confounders.

**Figure 3.**
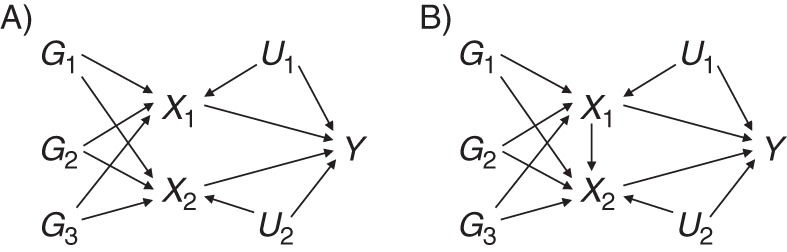
Causal directed acyclic graph illustrating multivariable Mendelian randomization in associations between variants *G*_1_, *G*_2_, and *G*_3_, risk factors *X*_1_ and *X*_2_, and outcome *Y*. Confounders *U*_1_ and *U*_2_ are assumed to be unknown. A) Risk factors are causally independent (no causal effects between *X*_1_ and *X*_2_); B) risk factors are causally dependent (*X*_1_ has a causal effect on *X*_2_).

In order to define and interpret causal effect estimates, we initially assume that the effect of each of the risk factors on the outcome is not mediated by another of the risk factors: We could intervene on each risk factor independently of all the other risk factors, and an intervention on one risk factor will not influence any other risk factor. We refer to such risk factors as “causally independent.” A causal directed acyclic graph corresponding to these assumptions with 3 genetic variants and 2 risk factors is presented in Figure 3A. We later relax the assumption of causal independence and allow causal effects between the risk factors, as in Figure 3B. In this paper, we assume that all associations are linear.

### Individual-level data: 2-stage least squares method

If individual-level data are available on the genetic variants, the risk factors, and the outcome, causal effects of the risk factors on the outcome can be estimated using a 2-stage least squares (2SLS) approach (14). The risk factors are regressed on the genetic variants in a multivariate linear regression (first stage; a multivariate multiple regression, since there are multiple dependent variables and multiple independent variables), and then the outcome is regressed linearly on the fitted values of each of the risk factors (second stage; a univariate multiple regression, since there is 1 dependent variable and multiple independent variables). An alternative model for the genetic association with the risk factor could be proposed (such as one including interaction terms), but a model which is additive and linear in the variants is used here for comparability with the summarized data methods considered in the next section. Although a sequential regression approach gives the correct point estimates, the use of 2SLS software (such as the *ivreg2* command in Stata (StataCorp LP, College Station, Texas) (15)) is recommended for estimation in practice to derive correct standard errors (16). Estimates from the method are valid even if the genetic variants are in linkage disequilibrium.

### Summarized data: likelihood-based method

If individual-level data are not available but rather we have summarized (aggregated) data on the beta coefficients and standard errors for the associations between the genetic variants and the risk factors and outcome from separate univariate regressions, then the causal effects of the risk factors on the outcome can be estimated using a likelihood-based method (17). For example, if there are 2 risk factors *X*_1_ and *X*_2_, each of which has no causal effect on the other, a multivariate normal distribution can be assumed for the beta coefficients representing the genetic associations with each of the risk factors *X*_1_ and *X*_2_ and the outcome *Y* from univariate linear regressions. Specifically, we assume that the estimate of association of genetic variant *j*, *j* = 1, …, *J*, with *X*_1_ is *X*_1*j*_ with standard error σ*_X_*_1*j*_, and similarly with *X*_2_ (*X*_2*j*_, standard error σ*_X_*_2*j*_) and with *Y* (*Y_j_*, standard error σ*_Yj_*):

(1) $$\left(\begin{array}{c} X_{1j}\\ X_{2j}\\ Y_{j} \end{array}\right)∼N_{3}\left(\left(\begin{array}{c} \xi_{1j}\\ \xi_{2j}\\ \beta_{1}\xi_{1j}+\beta_{2}\xi_{2j} \end{array}\right),\left(\begin{array}{ccc} \sigma_{X1j}^{2} & \rho_{12}\sigma_{X1j}\sigma_{X2j} & \rho_{1Y}\sigma_{X1j}\sigma_{Yj}\\ \rho_{12}\sigma_{X1j}\sigma_{X2j} & \sigma_{X2j}^{2} & \rho_{2Y}\sigma_{X2j}\sigma_{Yj}\\ \rho_{1Y}\sigma_{X1j}\sigma_{Yj} & \rho_{2Y}\sigma_{X2j}\sigma_{Yj} & \sigma_{Yj}^{2} \end{array}\right)\right).$$

Estimates of the causal effects of *X*_1_ and *X*_2_ on *Y* (β_1_ and β_2_) can be obtained by numerical maximization of this likelihood function or by Bayesian methods (18). If there are *K* risk factors, data on *K* + 1 beta coefficients and corresponding standard errors are required for each genetic variant (*X*_1*j*_, …, *X_Kj_*, *Y_j_*); there are *K*(*J* + 1) parameters in the model, and equation 1 shows a (*K* + 1)-variate normal distribution. If the outcome is binary and the beta coefficients for the genetic association with the outcome represent log relative risks or log odds ratios, then the causal effect estimates will represent log relative risks or log odds ratios, respectively. The model for the genetic associations with the outcome is linear in contributions from the genetic associations with the risk factors.

The parameters ρ_12_, ρ_1*Y*_, and ρ_2*Y*_ represent the correlations between the beta coefficients. These will be nonzero if the beta coefficients are derived from the same data. Although these correlations can only be estimated from individual-level data, they should be approximately equal to the observational correlations between the variables *X*_1_, *X*_2_, and *Y*. It is advisable to conduct a sensitivity analysis to assess the impact of these parameter values on the causal estimates. If data on the associations with the risk factors and outcome are obtained from separate data sources, the relevant correlations will be zero.

Because the likelihood function comprises contributions from each variant, it is necessary that the information on the causal parameters provided by each variant be independent. Therefore, the genetic variants used in a summarized data analysis must be uncorrelated (not in linkage disequilibrium); otherwise confidence intervals estimated by the method will be too narrow (17). If the genetic variants are in linkage disequilibrium and the correlations between the variants are known, then these correlations can be used in a modified likelihood-based model: The correlations between the genetic variants are the same as the correlations between the beta coefficients corresponding to the genetic variants. If all of the variants are correlated, then instead of a separate (*K* +1)-variate normal distribution for each of the *J* genetic variants, we can employ a *J*(*K* + 1)-variate normal distribution in equation 1 for all of the variants together.

### Summarized data: regression-based method

A further method which has been proposed for the analysis of summarized data is a linear regression-based approach, which gives estimates for each of the risk factors separately (19). This is performed in 2 stages. First, the beta coefficients for the genetic association with the outcome are regressed on the beta coefficients for the competing risk factors. Then the residuals from the first regression are regressed on the beta coefficients for the risk factor of interest.

For example, if there are 2 risk factors *X*_1_ and *X*_2_ and we want to estimate the effect of *X*_1_ on *Y*, the 2 stages are:

1. Regress the beta coefficients *Y*_1_, *Y*_2_, …, *Y_J_* on the beta coefficients *X*_21_, *X*_22_, …, *X*_2*J*_ to obtain residuals $\bar{Y}=Y-{\hat{\beta }}_{2}X_{2}$.
2. Regress the residuals ${\bar{Y}}_{1},{\bar{Y}}_{2},\ldots ,{\bar{Y}}_{J}$ on the beta coefficients *X*_11_, *X*_12_, …, *X*_1*J*_. The regression-based estimate is the regression coefficient for *X*_1_.

The intuitive rationale is that these residuals represent any causal effects that are not explained by the alternative risk factors but are potentially explained by the risk factor of interest. However, it is an ad hoc approach which has no clear theoretical basis and which ignores the uncertainty in the beta coefficients (20).

## EXAMPLE: CAUSAL EFFECTS OF LDL-C, HDL-C, AND TRIGLYCERIDES ON CHD RISK

The causal nature of the associations of various lipid fractions, including low-density lipoprotein cholesterol (LDL-C), high-density lipoprotein cholesterol (HDL-C), and triglycerides, with the risk of coronary heart disease (CHD) is an issue with important consequences for disease prevention and drug development strategies. Observational studies have shown associations of LDL-C with increased CHD risk, associations of HDL-C with decreased CHD risk, and a null association of triglycerides with CHD risk upon adjustment for a number of risk factors, including HDL-C and non-HDL-C concentrations, systolic blood pressure, and body mass index (21). However, a causal interpretation of these results may be misleading due to unmeasured confounding and the possibility that some of the covariates adjusted for lie on causal pathways, making their inclusion in a regression model inappropriate. Efforts to elucidate causal relationships using genetic variants in a Mendelian randomization approach have indicated that LDL-C plays a causal role in increasing the risk of CHD (22) and have suggested a null causal effect of HDL-C on CHD risk (23). However, the latter estimate had wide confidence intervals, because only a few variants—those not associated with other lipid fractions—were included in the analysis. The inability to find variants associated with triglycerides and not associated with LDL-C or HDL-C has precluded reliable Mendelian randomization investigations for triglycerides.

**Figure 4.**
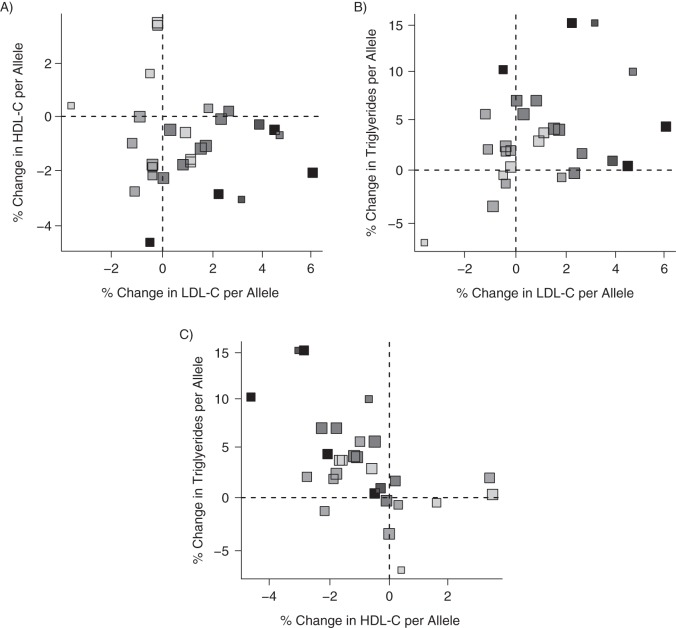
Associations of coronary heart disease (CHD) risk-increasing alleles of 28 genetic variants with all possible pairings of low-density lipoprotein cholesterol (LDL-C), high-density lipoprotein cholesterol (HDL-C), and triglycerides. Darker points correspond to stronger associations with CHD risk; larger points correspond to more precise estimates. Note that some points are overlapping.

**Figure 5.**
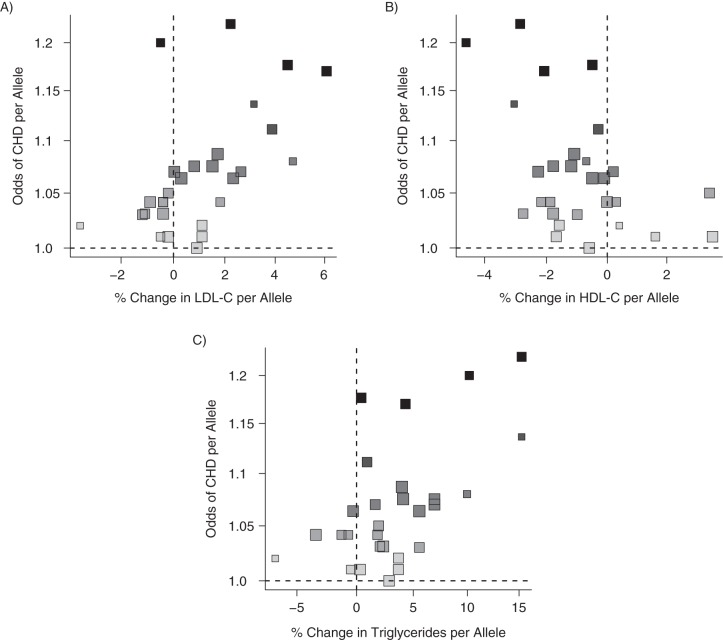
Associations of coronary heart disease (CHD) risk-increasing alleles of 28 genetic variants with odds of CHD and with low-density lipoprotein cholesterol (LDL-C) (A), high-density lipoprotein cholesterol (HDL-C) (B), and triglycerides (C). Darker points correspond to stronger associations with CHD risk; larger points correspond to more precise estimates. Note that some points are overlapping.

Here we address the question of the causal effects of LDL-C, HDL-C, and triglycerides on CHD risk by multivariable Mendelian randomization using published data. Waterworth et al. (24) reported genetic associations from univariate regression analyses of 28 genetic variants with log-transformed LDL-C, HDL-C, and triglyceride concentrations and with the log odds of CHD. Details on the variants and the β coefficients for the associations are given in Web Table 1. Figures 4 and 5 depict the associations of each of the variants with the lipid fractions and CHD risk.

We combine these beta coefficients in a multivariable Mendelian randomization analysis using the likelihood-based and regression-based methods. Estimates using the likelihood-based method were obtained in a Bayesian framework using WinBUGS (http://www.mrc-bsu.cam.ac.uk/bugs). Technical details on the analysis and the software code used are provided in Web Appendix 1. A sensitivity analysis for the values of the correlation parameters (ρ_1*Y*_, ρ_2*Y*_, …) is given in Web Table 2. Initially, we do not account for linkage disequilibrium between the genetic variants so that the analysis methods can be more directly compared.

Using the likelihood-based method, the multivariable Mendelian randomization analysis gives causal odds ratios and 95% credible intervals for CHD of 0.50 (95% credible interval (CrI): 0.40, 0.62) per 30% reduction in LDL-C, 1.22 (95% CrI: 0.91, 1.63) per 30% reduction in HDL-C, and 0.77 (95% CrI: 0.68, 0.87) per 30% reduction in triglycerides. This suggests that reductions in LDL-C and in triglycerides are causally protective against CHD. The causal effect for HDL-C is compatible with the null. The regression-based method produces rather different results: The corresponding odds ratios and 95% confidence intervals are 0.69 (95% confidence interval: 0.51, 0.92) for LDL-C, 1.25 (95% confidence interval: 0.90, 1.74) for HDL-C, and 0.92 (95% confidence interval: 0.82, 1.04) for triglycerides. In particular, the causal odds ratio for triglycerides from the regression-based method does not reach the conventional threshold of statistical significance. When linkage disequilibrium between the genetic variants is accounted for, the likelihood-based method gives odds ratios and 95% credible intervals of 0.52 (95% CrI: 0.42, 0.65) for LDL-C, 1.28 (95% CrI: 0.96, 1.71) for HDL-C, and 0.78 (95% CrI: 0.70, 0.86) for triglycerides.

## SIMULATION STUDY

In order to assess the statistical properties of the analysis methods used, we perform a simulation study. The setup corresponds to the example above.

We generate data for 30,000 individuals indexed by *i* on 3 risk factors (*X*_1_, *X*_2_, *X*_3_) and an outcome (*Y*) from the following data-generating model:

(2) $$\begin{array}{cc} x_{1i} & =\sum_{\;j=1}^{28}\;\alpha_{G1j}g_{ij}+\alpha_{U2}u_{2i}+\alpha_{U3}u_{3i}+\epsilon_{X1i}.\\ x_{2i} & =\sum_{\;j=1}^{28}\;\alpha_{G2j}g_{ij}+\alpha_{U1}u_{1i}+\alpha_{U3}u_{3i}+\epsilon_{X2i}.\\ x_{3i} & =\sum_{\;j=1}^{28}\;\alpha_{G3j}g_{ij}+\alpha_{U1}u_{1i}+\alpha_{U2}u_{2i}+\epsilon_{X3i}.\\ y_{i} & =\beta_{U1}u_{1i}+\beta_{U2}u_{2i}+\beta_{U3}u_{3i}+\beta_{1}x_{1i}\\ & \;+\beta_{2}x_{2i}+\beta_{3}x_{3i}+\epsilon_{Yi}.\\ g_{ij} & ∼\text{Binomial}(\text{2,}\;\text{0}.3)\;\text{independently}\;\text{for}\;\text{each}\;\\ & \;\;\;j=1,\ldots ,28.\\ u_{1i},u_{2i},u_{3i} & ∼N(0,1)\;\text{independently}.\\ \epsilon_{X1i},\epsilon_{X2i},\epsilon_{X3i},\epsilon_{Yi} & ∼N(0,1)\;\text{independently}. \end{array}$$

We set the genetic association parameters α*_G_*_1*j*_, α*_G_*_2*j*_, and α*_G_*_3*j*_ for *j* = 1, …, 28 to take the values shown in Web Table 1 for log-transformed LDL-C, HDL-C, and triglycerides, respectively, to be similar to the applied example. The instrumental variables *g_ij_* are drawn from binomial distributions, representing independent single-nucleotide polymorphisms with minor allele frequencies of 0.3. The causal effects β_1_, β_2_, and β_3_ are set to 0.3, 0, and −0.1, respectively. The variables *U*_1_, *U*_2_, *U*_3_ represent confounders, leading to correlations between *X*_1_, *X*_2_, *X*_3_ and *Y*. The parameters β*_U_*_1_, β*_U_*_2_, β*_U_*_3_ are each fixed at 0.3 throughout, and the parameters α*_U_*_1_, α*_U_*_2_, α*_U_*_3_ are varied to take the value 0.3 or −0.3, leading to 8 different scenarios. The mean *R*^2^ values, representing the proportion of variation in each risk factor explained by the 28 instrumental variables together, for *X*_1_, *X*_2_, and *X*_3_ are 0.6%, 0.5%, and 3.2%, respectively, corresponding to mean *F* statistics of 6.6, 5.2, and 35.4.

Estimates for 1,000 data sets generated in each of the 8 scenarios considered were derived using the 2SLS, likelihood-based, and regression-based methods. The Monte Carlo standard errors for the mean estimates were approximately 0.003, and for the power they were approximately 1%. The likelihood-based method was applied in a Bayesian framework using WinBUGS; technical details on the analyses are provided in Web Appendix 2.

Table 1 shows, for each scenario, the mean estimate, the mean standard error, the standard deviation of the estimates, and the statistical power to detect a nonzero effect at a nominal 5% significance level. For β_2_ = 0, the expected power is 5%. We see that the mean estimates from the 2SLS and likelihood-based methods are close to the true values, with some deviation depending on the direction of confounding. This may represent the effect of weak instrument bias, corresponding to the low *F* statistics above for *X*_1_ and *X*_2_ (25). The efficiencies of the 2SLS and likelihood-based methods are similar, despite the reliance of the likelihood-based method on only summarized data.

**Table 1.** Results From a Simulation Study of the Use of Multivariable Mendelian Randomization to Estimate 3 Causal Effects in Scenarios Without Causal Relationships Between Risk Factors^a^

α_U__1_	α_U__2_	α_U__3_	2-Stage Least Squares Method				Likelihood-Based Method				Regression-Based Method			
			Mean Estimate	Mean SE	SD of Estimates	Power, %^b^	Mean Estimate	Mean SE	SD of Estimates	Power, %^b^	Mean Estimate	Mean SE	SD of Estimates	Power, %^b^
*Estimate of β_1_ = 0.3*														
0.3	0.3	0.3	0.318	0.090	0.091	93.0	0.317	0.092	0.091	91.2	0.224	0.078	0.070	85.1
0.3	0.3	−0.3	0.290	0.090	0.091	88.8	0.290	0.092	0.092	86.9	0.208	0.078	0.070	77.7
0.3	−0.3	0.3	0.296	0.090	0.089	90.2	0.296	0.092	0.090	89.3	0.211	0.078	0.069	81.7
0.3	−0.3	−0.3	0.271	0.090	0.089	86.7	0.272	0.092	0.090	85.3	0.193	0.077	0.067	74.9
−0.3	0.3	0.3	0.329	0.091	0.092	93.1	0.328	0.092	0.093	91.7	0.234	0.079	0.073	85.9
−0.3	0.3	−0.3	0.305	0.090	0.090	92.1	0.304	0.092	0.091	91.0	0.220	0.078	0.069	85.0
−0.3	−0.3	0.3	0.309	0.090	0.086	93.6	0.309	0.092	0.086	92.1	0.221	0.079	0.068	84.4
−0.3	−0.3	−0.3	0.283	0.090	0.088	88.1	0.283	0.092	0.089	86.7	0.204	0.078	0.068	77.9
*Estimate of β_2_ = 0*														
0.3	0.3	0.3	0.055	0.110	0.111	7.7	0.054	0.113	0.111	7.8	0.035	0.085	0.069	4.2
0.3	0.3	−0.3	0.010	0.111	0.113	5.9	0.010	0.114	0.114	6.1	0.007	0.085	0.069	2.2
0.3	−0.3	0.3	0.040	0.111	0.112	6.2	0.040	0.113	0.113	6.4	0.027	0.085	0.070	2.8
0.3	−0.3	−0.3	0.001	0.111	0.111	4.1	0.001	0.114	0.112	4.8	0.002	0.085	0.069	2.2
−0.3	0.3	0.3	0.001	0.111	0.112	3.9	0.001	0.114	0.113	4.7	−0.001	0.085	0.069	1.7
−0.3	0.3	−0.3	−0.050	0.111	0.107	6.6	−0.049	0.113	0.108	6.8	−0.033	0.085	0.067	2.8
−0.3	−0.3	0.3	−0.008	0.111	0.113	5.6	−0.007	0.114	0.115	6.0	−0.006	0.086	0.071	2.6
−0.3	−0.3	−0.3	−0.045	0.110	0.111	7.6	−0.045	0.112	0.112	7.1	−0.031	0.085	0.070	2.9
*Estimate of β_3_ = −0.1*														
0.3	0.3	0.3	−0.087	0.047	0.045	47.4	−0.087	0.047	0.045	46.4	−0.039	0.033	0.023	13.0
0.3	0.3	−0.3	−0.090	0.047	0.049	49.3	−0.090	0.048	0.049	49.8	−0.041	0.033	0.024	16.7
0.3	−0.3	0.3	−0.090	0.047	0.045	49.2	−0.090	0.047	0.046	47.8	−0.041	0.033	0.023	14.3
0.3	−0.3	−0.3	−0.094	0.047	0.047	52.0	−0.094	0.048	0.047	49.7	−0.043	0.033	0.023	16.9
−0.3	0.3	0.3	−0.106	0.047	0.048	61.2	−0.106	0.048	0.048	58.6	−0.049	0.033	0.025	25.8
−0.3	0.3	−0.3	−0.111	0.047	0.046	66.2	−0.110	0.048	0.047	62.6	−0.052	0.033	0.024	28.2
−0.3	−0.3	0.3	−0.105	0.047	0.044	63.5	−0.105	0.048	0.045	59.7	−0.049	0.034	0.023	24.8
−0.3	−0.3	−0.3	−0.111	0.047	0.046	66.4	−0.110	0.047	0.047	63.1	−0.052	0.033	0.024	28.9

Abbreviations: SD, standard deviation; SE, standard error.

^a^ Three analytical methods (2-stage least squares, likelihood-based, and regression-based) were used to estimate the causal effects of *X*_1_ on *Y* (β_1_ = 0.3), *X*_2_ on *Y* (β_2_ = 0), and *X*_3_ on *Y* (β_3_ = –0.1).

^b^ Empirical power to detect a causal effect at a nominal 5% significance level.

In contrast, estimates from the regression-based method are biased, although they appear to give approximately valid inferences for the presence of a causal effect under the null. However, the power, especially the power to estimate β_3_, is much lower than that from the other methods. We therefore recommend the likelihood-based method for use in practice when summarized data are available.

### Causal relationships between risk factors

In order to investigate the performance of the methods when there are causal relationships between the risk factors, we repeated the simulation but replaced the first line with

$$\begin{array}{c} x_{1i}=\sum_{\;j=1}^{28}\;\alpha_{G1j}g_{ij}+\alpha_{U2}u_{2i}+\alpha_{U3}u_{3i}+\alpha_{X2}x_{2i}+\alpha_{X3}x_{3i}+\epsilon_{X1i}. \end{array}$$

The additional terms α*_X_*_2_*x*_2*i*_ and α*_X_*_3_*x*_3*i*_ represent causal effects of *X*_2_ and *X*_3_ (which were evaluated first) on *X*_1_. We set α*_U_*_1_, α*_U_*_2_, and α*_U_*_3_ equal to 0.3 (the first scenario considered above) and took 9 values of the parameters α*_X_*_2_ and α*_X_*_3_ (Table 2). All other parameters were taken as in the original simulation study.

**Table 2.** Results From a Simulation Study of the Use of Multivariable Mendelian Randomization to Estimate 3 Causal Effects in Scenarios With Causal Relationships Between Risk Factors^a^

α*_X_*_2_	α*_X_*_3_	2-Stage Least Squares Method			Likelihood-Based Method			Regression-Based Method		
		β_1_	β_2_	β_3_	β_1_	β_2_	β_3_	β_1_	β_2_	β_3_
0	0	0.318	0.055	−0.087	0.317	0.054	−0.087	0.224	0.035	−0.039
0.5	0	0.322	0.038	−0.090	0.321	0.038	−0.090	0.229	0.020	−0.041
−0.5	0	0.318	0.059	−0.089	0.318	0.058	−0.089	0.164	0.034	−0.041
0	0.5	0.317	0.046	−0.097	0.316	0.045	−0.097	0.064	0.030	−0.016
0	−0.5	0.321	0.048	−0.079	0.320	0.048	−0.079	0.192	0.030	−0.037
0.5	0.5	0.316	0.041	−0.097	0.315	0.041	−0.097	0.077	0.021	−0.016
−0.5	0.5	0.318	0.060	−0.098	0.318	0.060	−0.098	0.049	0.036	−0.016
0.5	−0.5	0.318	0.042	−0.080	0.317	0.042	−0.081	0.125	0.022	−0.038
−0.5	−0.5	0.317	0.057	−0.079	0.316	0.056	−0.080	0.240	0.034	−0.037

^a^ Three analytical methods (2-stage least squares, likelihood-based, and regression-based) were used to estimate direct causal effects of *X*_1_ on *Y* (β_1_ = 0.3), *X*_2_ on *Y* (β_2_ = 0), and *X*_3_ on *Y* (β_3_ = –0.1).

Table 2 shows the mean estimates of the causal parameters derived from each of the methods. Aside from the regression-based method, which produces widely varying results, we see that the estimates do not change substantially as the parameters vary. This indicates that the 2SLS and likelihood-based methods estimate the direct causal effect of each risk factor on the outcome, not including paths operating via the other risk factors. This can lead to misleading conclusions about the total effects of the variables. For example, when α*_X_*_2_ = 0 and α*_X_*_3_ = 0.5, the total causal effect of *X*_3_ on *Y* is $\beta_{3}+\alpha_{X1}\beta_{1}=-0.1+0.5\times 0.3=0.05$ (including the path operating via *X*_1_). The mean estimates from the 2SLS and likelihood-based methods are in the opposite direction of the true total effect.

The differences between the estimated values of β_1_, β_2_, and β_3_ in Table 2 and their true values can be attributed to weak instrument bias. Weak instrument scenarios will be common in multivariable Mendelian randomization, as it is necessary to use multiple instrumental variables to estimate the different causal effects. If the genetic associations with the risk factors and with the outcome are measured in the same data set, this will lead to bias in the direction of the observational association (25), whereas if the genetic associations with the risk factors and with the outcome come from different sources (known as a 2-sample instrumental-variable analysis), the bias will be in the direction of the null (26). To demonstrate this, we repeat the simulation study outlined in Web Appendix 3 with fewer nonweak instrumental variables. The mean estimates of the causal effect parameters are very close to their true values (Web Table 3). In this simulation context, we also explore the impact of interactions between genetic variants in their effects on the risk factors. The likelihood-based method is robust to these misspecifications of the analysis model (Web Table 4). We also investigate a modification of the 2SLS method referred to as “sequential adjustment” by Holmes et al. (27), in which the causal effects of each of the risk factors are estimated in turn, and alternative risk factors are adjusted for as if they are confounders. Web Tables 5 and 6 indicate that substantial bias in the sequential adjustment method is evident even under the null, and its direction depends on the unknown confounders.

A nonzero causal estimate from a multivariable Mendelian randomization approach when there are causal relationships between the risk factors implies that the variable is an independent causal risk factor, in the sense that an intervention on the variable keeping the other risk factors constant (the controlled direct effect) would affect the outcome. However, the magnitude of the causal estimate may not represent the total causal effect of the variable on the outcome.

## DISCUSSION

In this paper, we have introduced multivariable Mendelian randomization, an important and practically relevant extension of the Mendelian randomization paradigm for estimation of causal effects using genetic variants associated with more than 1 risk factor. For a valid analysis, the variants must satisfy a set of assumptions that are similar to those for an instrumental variable in conventional Mendelian randomization but are modified to take account of the multiple risk factors. A multivariable Mendelian randomization analysis may be beneficial where genetic variants are associated with several related risk factors, such as in the example with lipid fractions. It permits causal evaluation of a risk factor even if no variants are uniquely associated with it, as for triglycerides.

There are several limitations to this approach, many of which are shared with conventional Mendelian randomization (28, 29). The specific association of a genetic variant with a single risk factor may be a reasonable assumption if the function of the genetic region where the variant is located is well-characterized. The assumption of an exclusive association between genetic variants and a set of risk factors is unlikely unless the risk factors have strong biological associations. However, if they are strongly associated, an assumption that the risk factors are causally independent is less plausible. Weak instrument bias, a phenomenon by which instrumental-variable estimates using variants not strongly associated with the risk factor of interest are biased, may be substantial if large numbers of genetic variants are used (30), as may be necessary in a multivariable Mendelian randomization experiment. The effects of the risk factors on the outcome are assumed to be linear. While some researchers do not view this as a crucial assumption, citing the interpretation of an instrumental-variable estimate as an average causal effect (6, 31), others have shown that departures from linearity can affect the findings of an instrumental-variable analysis (32). Our preference is to take a less literal view of causal estimates and to emphasize the outcome of a Mendelian randomization analysis as reflecting testing of a causal effect, rather than necessarily estimation of a causal parameter. From this perspective, while the linearity assumption is important, it is less important than the other instrumental-variable assumptions.

Although multivariable Mendelian randomization is able to allow for genetic variants with “measured” pleiotropic associations, under the assumptions discussed in this paper, it is unable to deal with unmeasured or unknown pleiotropy. If an apparent causal finding is dependent on the association of a small number of variants with the outcome, then the result may plausibly be due to pleiotropic variants rather than being a true causal effect. However, if several variants in different genetic regions all demonstrate consistent associations with the outcome, then it is perhaps unlikely that all of these associations reflect pleiotropic mechanisms (33). In the case of our applied example, we constructed a lipid risk score for each variant by multiplying the genetic associations with each lipid fraction by the estimate of the lipid fraction's causal effect on CHD risk; details are given in Web Appendix 4. Web Figure 2 displays the lipid risk score plotted against the log odds ratio of CHD risk for each variant. Aside from variant rs2304130, it seems that the estimated causal effect of the lipid fractions on CHD risk is consistent across variants, and so unmeasured pleiotropy is unlikely to explain the causal effects.

We performed the applied analysis for the causal effects of lipid fractions on CHD risk in this paper at face value, assuming that the instrumental-variable assumptions were satisfied. In reality, the assumptions that there were only 3 lipid categories and that the effects of the genetic variants were restricted to these lipid fractions are oversimplifications. Some lipid fractions (e.g., intermediate-density lipoprotein cholesterol) were omitted from the analysis, and the variability of particle size within the categories was ignored (34). An assumption that the causal effect of triglycerides on CHD risk is independent of the effects of LDL-C and HDL-C may not be satisfied, particularly as evidenced by the attenuation of the observational association of triglycerides with CHD risk upon adjustment for HDL-C and non-HDL-C (21) and studies of the apolipoprotein A5 gene (*APOA5*) (35). Our simulations above have shown that in the case of causal effects between risk factors, estimates represent the direct causal effect of each risk factor on the outcome by a pathway that is not operating via the other risk factors. This may not equal the total causal effect of the risk factor, but it provides important evidence on the independent causal effect of the risk factor. Finally, our estimated causal odds ratio for a 30% decrease in LDL-C was surprisingly large in comparison with not only estimates of the effect of statin usage, which also reduces LDL-C levels by approximately 30% (22), but also a Mendelian randomization analysis that included variants solely associated with LDL-C (17). A nuanced interpretation of Mendelian randomization estimates, and of multivariable Mendelian randomization estimates in particular, is required in the light of the uncertainty of the underlying assumptions in any applied analysis. These aspects are considered in more detail elsewhere (20).

In conclusion, these findings provide some evidence of a causal effect of triglyceride-related pathways on CHD risk independent of the effects of LDL-C and HDL-C, but the weight of evidence attributed to the findings is a matter of interpretation depending on the degree of validity attributed to the instrumental-variable assumptions.

***Note added in proof:*** *While our manuscript was in press, we discovered a simple modification of the discussed regression-based method that uses available statistical software to produce estimates with much better theoretical and statistical properties. See Burgess et al. (36) for further information.*

## Supplementary Material

Web Material
