# Supplementary material for: Multivariable Mendelian Randomization: The Use of Pleiotropic Genetic Variants to Estimate Causal Effects
Source: Am J Epidemiol. 2015 Jan 27;181(4):251–60. doi: 10.1093/aje/kwu283 (PMC4325677; doi:10.1093/aje/kwu283)
Supplement: Web Material [file supp_181_4_251__index.html]

Multivariable Mendelian Randomization: The Use of Pleiotropic Genetic Variants to Estimate Causal Effects — Multivariable Mendelian Randomization: The Use of Pleiotropic Genetic Variants to Estimate Causal Effects — Web Material 

# Multivariable Mendelian Randomization: The Use of Pleiotropic Genetic Variants to Estimate Causal Effects

## Web Material

Web Material

**Files in this Data Supplement:**

- Web Material - Pdf file
