# Supplementary material for: Multivariable Mendelian Randomization: The Use of Pleiotropic Genetic Variants to Estimate Causal Effects
Source: Am J Epidemiol. 2015 Jan 27;181(4):251–60. doi: 10.1093/aje/kwu283 (PMC4325677; doi:10.1093/aje/kwu283)
Supplement: Web Material [file supp_kwu283_kwu283supp.pdf]

Factorial randomized trial		Randomization of A	
		Control	Treatment A
Randomization of B	Control	Incidence under usual care	Incidence under intervention A
	Treatment B	Incidence under intervention B	Incidence under interventions A and B

Factorial Mendelian randomization		Genetic variant 1	
		Genotype AA	Genotype aa
Genetic variant 2	Genotype BB	AABB (LDL-C, TG usual levels)	aaBB (LDL-C much higher, TG slightly higher)
	Genotype bb	AAbb (LDL-C slightly higher, TG much higher)	aabb (LDL-C much higher, TG much higher)

Web Figure 1: Analogy between a factorial randomized trial with two treatments (A and B) and multivariable Mendelian randomization with two genetic variants (1 and 2). The minor allele of genetic variant 1 (a) has a large effect on LDL-C and a modest effect on triglycerides; the minor allele of genetic variant 2 (b) has a large effect on triglycerides and a modest effect on LDL-C.

Abbreviations: LDL-C = low-density lipoprotein cholesterol, TG = triglycerides.

Web Appendix 1

Supplementary methods for applied example

Web Table 1 gives the estimates of the associations between each of 28 genetic variants and low-density lipoprotein cholesterol (LDL-C), triglycerides, high-density lipoprotein cholesterol (HDL-C), and the log odds ratio of coronary heart disease (CHD) reported by Waterworth et al. [1].

Estimates for the analysis from the likelihood-based method were obtained in a Bayesian framework using WinBUGS (http://www.mrc-bsu.cam.ac.uk/bugs). Direct maximization of the likelihood is impractical in this case, as there are $28 \times 3 + 3 = 87$ parameters to optimize over. In the likelihood function, the correlation between the beta-coefficients for LDL-C and HDL-C was taken as -0.1, for LDL-C and triglycerides 0.2, for LDL-C and CHD risk 0.1, for HDL-C and triglycerides -0.1, for HDL-C and CHD risk -0.1, and for triglycerides and CHD risk 0.1. A sensitivity analysis for these values is given in Web Table 2. Normal priors with mean 0 and variance 10^2 are used for each of the unknown parameters. The WinBUGS code used to implement the likelihood-based analysis is:

```
model {
 beta1 ~ dnorm(0, 0.01) # prior for causal effect of LDL-C
 beta2 ~ dnorm(0, 0.01) # prior for causal effect of HDL-C
 beta3 ~ dnorm(0, 0.01) # prior for causal effect of triglycerides

 for (i in 1:28) {      # i indexes the 28 variants
  Tau0[i,1:4,1:4] <- inverse(Sigma0[i,1:4,1:4])
    # Sigma0 is the variance-covariance matrix for the multivariate
    # distribution of the beta-coefficients
```

```
  x[i,1:4] ~ dmnorm(xi[i,1:4], Tau0[i,1:4,1:4])
    # x[i, 1:4] are the beta-coefficients for genetic variant i
    # with LDL-C, HDL-C, triglycerides, and CHD risk
  xi[i,1] ~ dnorm(0, 0.01)
    # prior for mean association of genetic variant i with LDL-C
  xi[i,2] ~ dnorm(0, 0.01)
    # prior for mean association of genetic variant i with HDL-C
  xi[i,3] ~ dnorm(0, 0.01)
    # prior for mean association of genetic variant i with triglycerides
  xi[i,4] <- beta1*xi[i,1] + beta2*xi[i,2] + beta3*xi[i,3]
    # mean association of genetic variant i with CHD risk
  }
 }
```

The R code for the regression-based based analysis is:

```
for (j in 1:28) {
 bx1   = lm(x1~gene[,j])$coef[2]
 bx2   = lm(x2~gene[,j])$coef[2]
 bx3   = lm(x3~gene[,j])$coef[2]
 by    = lm(y ~gene[,j])$coef[2]
 bx1se = summary(lm(x1~gene[,j]))$coef[2,2]
 bx2se = summary(lm(x2~gene[,j]))$coef[2,2]
 bx3se = summary(lm(x3~gene[,j]))$coef[2,2]
 byse  = summary(lm(y ~gene[,j]))$coef[2,2]
 }
beta1 = lm(lm(by~bx2+bx3)$res~bx1)$coef[2]
beta2 = lm(lm(by~bx1+bx3)$res~bx2)$coef[2]
beta3 = lm(lm(by~bx1+bx2)$res~bx3)$coef[2]
beta1se = summary(lm(lm(by~bx2+bx3)$res~bx1))$coef[2,2]
beta2se = summary(lm(lm(by~bx1+bx3)$res~bx2))$coef[2,2]
beta3se = summary(lm(lm(by~bx1+bx2)$res~bx3))$coef[2,2]
```

Estimates of the correlations between genetic variants were taken from the 1000 Genomes Pilot 1 dataset and obtained from the SNP Annotation and Proxy Search (SNAP; http://www.broadinstitute.org/mpg/snap/ldsearchpw.php).

Sensitivity analysis for correlation parameters in likelihood-based method

In order to assess the sensitivity of the causal estimates in the applied example to the choice of correlation parameters, we performed a sensitivity analysis, estimating the causal effects of LDL-C, HDL-C and triglycerides on CHD risk for a number of different choices of the parameters. Web Table 2 displays the causal estimates for the parameter values used in the main analysis (shown in italics), as well as for values 2, 1.5, 0.5, 0, and -1 times these values. We see that the causal estimates and standard errors are fairly robust to different values of these parameters.

Genetic variant	X_L (SE)	X_T (SE)	X_H (SE)	Y (SE)
rs11206510	0.026 (0.004)	0.016 (0.008)	0.002 (0.004)	0.068 (0.029)
rs660240	-0.044 (0.004)	-0.004 (0.008)	0.005 (0.004)	-0.162 (0.030)
rs515135	-0.038 (0.004)	-0.009 (0.008)	0.003 (0.004)	-0.105 (0.031)
rs12916	-0.023 (0.003)	0.003 (0.006)	0.001 (0.003)	-0.062 (0.024)
rs2954021	-0.017 (0.003)	-0.039 (0.006)	0.011 (0.003)	-0.083 (0.022)
rs1558861	-0.031 (0.006)	-0.142 (0.012)	0.031 (0.006)	-0.128 (0.067)
rs2738459	-0.018 (0.004)	0.007 (0.007)	-0.003 (0.004)	-0.041 (0.037)
rs10401969	0.046 (0.007)	0.095 (0.013)	-0.007 (0.006)	0.077 (0.054)
rs4420638	0.059 (0.004)	0.042 (0.008)	-0.021 (0.004)	0.157 (0.031)
rs10489615	0.004 (0.003)	-0.023 (0.006)	0.018 (0.003)	-0.030 (0.024)
rs11902417	0.011 (0.004)	0.036 (0.007)	-0.017 (0.003)	0.010 (0.028)
rs325	-0.005 (0.005)	0.097 (0.010)	-0.047 (0.005)	0.182 (0.040)
rs3890182	0.004 (0.005)	0.013 (0.009)	0.022 (0.004)	-0.041 (0.034)
rs964184	0.022 (0.005)	0.142 (0.009)	-0.029 (0.004)	0.199 (0.034)
rs9943753	-0.005 (0.004)	-0.005 (0.007)	0.016 (0.003)	0.010 (0.038)
rs261334	-0.002 (0.004)	0.019 (0.007)	0.034 (0.004)	0.049 (0.029)
rs9989419	-0.002 (0.003)	0.003 (0.006)	0.035 (0.003)	0.010 (0.025)
rs12449157	0.004 (0.004)	-0.018 (0.008)	0.019 (0.004)	-0.041 (0.032)
rs2156552	0.011 (0.004)	-0.020 (0.008)	0.028 (0.004)	-0.030 (0.032)
rs1168013	0.009 (0.003)	0.035 (0.007)	0.001 (0.003)	-0.041 (0.024)
rs6544366	-0.011 (0.004)	-0.036 (0.007)	0.016 (0.003)	-0.020 (0.028)
rs1260333	-0.003 (0.003)	-0.054 (0.006)	0.005 (0.003)	-0.062 (0.022)
rs1178979	-0.012 (0.004)	0.054 (0.008)	-0.010 (0.004)	0.030 (0.030)
rs10105606	0.003 (0.003)	0.067 (0.006)	-0.023 (0.003)	0.068 (0.024)
rs2954029	-0.015 (0.003)	-0.040 (0.006)	0.012 (0.003)	-0.073 (0.022)
rs4938303	-0.008 (0.004)	-0.067 (0.007)	0.018 (0.003)	-0.073 (0.025)
rs16965220	0.009 (0.003)	0.028 (0.006)	-0.006 (0.003)	0.000 (0.026)
rs2304130	-0.036 (0.007)	-0.070 (0.013)	0.004 (0.006)	0.020 (0.065)

Web Table 1: Association of 28 genetic variants with log-transformed low-density lipoprotein cholesterol (X_L), log-transformed triglycerides (X_T), log-transformed high-density lipoprotein cholesterol (X_H) and log odds ratio of coronary heart disease (Y) with standard errors (SE) taken from Waterworth et al. [1]

ρ_{LH}	ρ_{LT}	ρ_{LY}	ρ_{HT}	ρ_{HY}	ρ_{TY}	β_{LDL-C} (SE)	β_{HDL-C} (SE)	β_{TG} (SE)
-0.2	0.4	0.2	-0.2	-0.2	0.2	1.96 (0.31)	-0.53 (0.39)	0.74 (0.16)
-0.15	0.3	0.15	-0.15	-0.15	0.15	1.96 (0.31)	-0.55 (0.40)	0.74 (0.17)
-0.1	*0.2*	*0.1*	*-0.1*	*-0.1*	*0.1*	*1.96 (0.32)*	*-0.56 (0.41)*	*0.73 (0.17)*
-0.05	0.1	0.05	-0.05	-0.05	0.05	1.97 (0.32)	-0.57 (0.42)	0.73 (0.17)
0	0	0	0	0	0	1.97 (0.33)	-0.58 (0.42)	0.73 (0.18)
0.1	-0.2	-0.1	0.1	0.1	-0.1	1.97 (0.34)	-0.59 (0.44)	0.73 (0.18)

Web Table 2: Sensitivity analysis for the correlation parameters between beta-coefficients for genetic associations with low-density lipoprotein cholesterol (L), high-density lipoprotein cholesterol (H), triglycerides (T) and CHD risk (Y) in likelihood-based method for multivariable Mendelian randomization (original analysis shown in italics)

Web Appendix 2

Supplementary methods for simulation study

For the simulation studies, in the likelihood-based method, we regard the mean and standard deviation of the posterior distribution as the 'estimate' and 'standard error', and the 2.5th to 97.5th percentile range as the '95% confidence interval'. The observational correlations between the variables estimated in the individual-level data were used for the correlation parameters. Normal priors with mean zero and variance 10^2 were used for each of the unknown parameters.

Web Appendix 3

Additional simulation studies with non-weak instrumental variables

Using non-weak instrumental variables

In order to show that the differences between estimates in Tables 1 and 2 from the main text and the true values of the parameters for the two-stage least squares (2SLS) and likelihood-based methods are due to weak instruments, we repeat the simulation but using only 5 instrumental variables having strong associations with the risk factor. The data-generating model is:

$$\begin{aligned}
x_{1i} &= \sum_{j=1}^{5} \alpha_{G1j} g_{ij} + \alpha_{U2} u_{2i} + \alpha_{U3} u_{3i} + \alpha_{X2} x_{2i} + \alpha_{X3} x_{3i} + \epsilon_{X1i} \qquad (3)\\
x_{2i} &= \sum_{j=1}^{5} \alpha_{G2j} g_{ij} + \alpha_{U1} u_{1i} + \alpha_{U3} u_{3i} + \epsilon_{X2i}\\
x_{3i} &= \sum_{j=1}^{5} \alpha_{G3j} g_{ij} + \alpha_{U1} u_{1i} + \alpha_{U2} u_{2i} + \epsilon_{X3i}\\
y_i &= \beta_{U1} u_{1i} + \beta_{U2} u_{2i} + \beta_{U3} u_{3i} + \beta_1 x_{1i} + \beta_2 x_{2i} + \beta_3 x_{3i} + \epsilon_{Yi}\\
g_{ij} &\sim \text{Binomial}(2, 0.3) \text{ independently for each } j = 1, \ldots, 5\\
u_{1i}, u_{2i}, u_{3i} &\sim \mathcal{N}(0, 1) \text{ independently}\\
\epsilon_{X1i}, \epsilon_{X2i}, \epsilon_{X3i}, \epsilon_{Yi} &\sim \mathcal{N}(0, 1) \text{ independently}
\end{aligned}$$

The coefficients α_{Gkj} for risk factor k and genetic variant j are taken as:

Genetic variant (j)	α_{G1j}	α_{G2j}	α_{G3j}
1	0.1	0.3	0.5
2	0.2	0.4	0.1
3	0.3	0.5	0.2
4	0.4	0.1	0.3
5	0.5	0.2	0.4

We set $\alpha_{U1}, \alpha_{U2}, \alpha_{U3} = 0.3$ and take 9 values of the parameters α_{X2} and α_{X3} as in the second set of simulations in the main paper (the section 'Causal relationships between risk factors'). All other parameters are taken as in the main paper. The average F statistics for the associations of the genetic variants with each of risk factors are around 1180.

Results are displayed in Web Table 3. We see that there is much less difference between the estimated and true values of the causal effect parameters for the two-stage least squares and likelihood-based methods with non-weak instrumental variables.

		Two-stage least squares			Likelihood-based		
α_{X2}	α_{X3}	β_1	β_2	β_3	β_1	β_2	β_3
0	0	0.299	0.000	-0.099	0.299	0.000	-0.099
0.5	0	0.300	0.000	-0.099	0.300	0.000	-0.100
-0.5	0	0.300	0.000	-0.100	0.300	0.000	-0.100
0	0.5	0.299	0.001	-0.100	0.299	0.001	-0.100
0	-0.5	0.301	0.000	-0.102	0.301	0.000	-0.102
0.5	0.5	0.301	-0.001	-0.100	0.301	-0.001	-0.100
-0.5	0.5	0.301	0.000	-0.100	0.301	0.000	-0.100
0.5	-0.5	0.300	0.000	-0.100	0.300	0.000	-0.100
-0.5	-0.5	0.299	0.000	-0.100	0.299	0.000	-0.100

Web Table 3: Mean estimates of the causal effects of X_1 (true direct effect $\beta_1 = 0.3$), X_2 ($\beta_2 = 0$), and X_3 ($\beta_3 = -0.1$) using two-stage least squares and likelihood-based methods from simulation study of multivariable Mendelian randomization with causal relationships between the risk factors and non-weak genetic instrumental variables

Allowing interaction terms in the genetic associations with the risk factors

To investigate the impact of interactions between the effects of genetic variants on estimates from the two-stage least squares and likelihood-based methods, we extend the above simulation (3) by introducing interaction terms in the genetic associations with the risk factors into the data-generating model:

$$
\begin{aligned}
x_{1i} &= \sum_{j=1}^{5} \alpha_{G1j} g_{ij} + \sum_{j_1>j_2} \alpha_{G1j_1j_2} g_{ij_1} g_{ij_2} + \alpha_{U2} u_{2i} + \alpha_{U3} u_{3i} + \epsilon_{X1i} \\
x_{2i} &= \sum_{j=1}^{5} \alpha_{G2j} g_{ij} + \sum_{j_1>j_2} \alpha_{G2j_1j_2} g_{ij_1} g_{ij_2} + \alpha_{U1} u_{1i} + \alpha_{U3} u_{3i} + \epsilon_{X2i} \\
x_{3i} &= \sum_{j=1}^{5} \alpha_{G3j} g_{ij} + \sum_{j_1>j_2} \alpha_{G3j_1j_2} g_{ij_1} g_{ij_2} + \alpha_{U1} u_{1i} + \alpha_{U2} u_{2i} + \epsilon_{X3i}
\end{aligned}
$$

Effects between the risk factors (α_{X2}, α_{X3}) are set to zero so that the risk factors are causally independent. The interaction terms $\alpha_{G1j_1j_2}, \alpha_{G2j_1j_2}, \alpha_{G3j_1j_2}$ are drawn from a normal distribution with mean $\alpha_{G\times}$ and variance $\sigma_{\alpha_{G\times}}$. We take $\sigma_{\alpha_{G\times}} = 0.1$ and consider four different values of $\alpha_{G\times} = 0.1, 0.05, -0.05, -0.1$. We analyze the data using the two-stage least squares and likelihood-based methods as previously, not including the interaction terms in the analysis model.

Results are displayed in Web Table 4. Estimates do not change substantially despite large interaction terms (compared with the main effect terms) resulting in misspecification of the analysis models. We see that the two-stage least squares and likelihood-based methods are robust to interactions between the genetic variants in their associations with the risk factors, even if these interactions are ignored in the analysis model.

	Two-stage least squares			Likelihood-based		
$\alpha_{G\times}$	β_1	β_2	β_3	β_1	β_2	β_3
0.1	0.300	0.000	-0.100	0.301	0.000	-0.100
0.05	0.298	0.000	-0.098	0.299	0.000	-0.098
-0.05	0.300	0.002	-0.097	0.300	0.002	-0.097
0.1	0.302	0.002	-0.100	0.302	0.002	-0.101

Web Table 4: Mean estimates of the causal effects of X_1 (true direct effect $\beta_1 = 0.3$), X_2 ($\beta_2 = 0$), and X_3 ($\beta_3 = -0.1$) using two-stage least squares and likelihood-based methods from simulation study of multivariable Mendelian randomization with non-weak genetic instrumental variables and interactions in the data-generating model for the genetic associations with the risk factors

Sequential adjustment method

We additionally consider a method for dealing with multiple risk factors referred to by Holmes et al. [2] as a sequential adjustment method. This is a 2SLS instrumental variable analysis for each of the risk factors in turn with adjustment for the other risk factors in the regression models. We perform two versions of this method. In the first version of the method, adjustment for the other risk factors is made only in the second-stage regression model; in the second version, adjustment is made in both regression stages. For example, in the second version of the method, if there are three risk factors X_1, X_2 and X_3, then the estimate for X_1 is obtained by first regressing X_1 on the IV(s), X_2 and X_3, and then regressing Y on the fitted values of X_1 from the first-stage regression, X_2 and X_3. Both versions of the analysis are performed as it is not clear which version of the method was chosen by the original investigators in Holmes et al. [2]. To more closely mimic the method performed by the original authors, we also performed the methods using a weighted allele score in place of the individual genetic variants [3]. Weights were taken as the true coefficients for the risk factor under analysis in the data-generating model.

We consider 8 sets of parameter values for $\alpha_{U1}, \alpha_{U2}, \alpha_{U3} = \pm 0.3$ and set $\alpha_{X2} = 0$ and $\alpha_{X3} = 0$ as in the initial set of simulations in the main paper. All other parameters are taken as in the main paper.

Results are given in Web Table 5 for the sequential adjustment methods using the individual variants as instrumental variables, and in Web Table 6 for the sequential adjustment methods using the allele scores as instrumental variables, with estimates from the 2SLS method using all of the risk factors as in the main body of the paper presented in each case for comparison. We see that, while the 2SLS method seems to give unbiased estimates of the causal parameters, that the estimates from the sequential adjustment method are biased, and not consistent in direction even under

the null.

			Two-stage least squares			Sequential adjustment (1)			Sequential adjustment (2)		
α_{U1}	α_{U2}	α_{U3}	β_1	β_2	β_3	β_1	β_2	β_3	β_1	β_2	β_3
0.3	0.3	0.3	0.299	0.000	-0.099	0.070	-0.181	-0.290	0.094	-0.179	-0.306
0.3	0.3	-0.3	0.300	0.000	-0.100	0.270	-0.090	-0.198	0.300	-0.089	-0.213
0.3	-0.3	0.3	0.300	0.000	-0.100	0.179	0.000	-0.181	0.207	0.000	-0.193
0.3	-0.3	-0.3	0.299	0.001	-0.100	0.379	0.091	-0.090	0.414	0.090	-0.100
-0.3	0.3	0.3	0.301	0.000	-0.102	0.161	-0.091	-0.091	0.186	-0.090	-0.101
-0.3	0.3	-0.3	0.301	-0.001	-0.100	0.362	0.000	0.002	0.393	0.000	-0.007
-0.3	-0.3	0.3	0.301	-0.001	-0.100	0.270	0.091	0.019	0.300	0.089	0.014
-0.3	-0.3	-0.3	0.300	0.000	-0.100	0.470	0.181	0.110	0.506	0.178	0.106

Web Table 5: Mean estimates of the causal effects of X_1 (true effect $\beta_1 = 0.3$), X_2 ($\beta_2 = 0$), and X_3 ($\beta_3 = -0.1$) using three different methods from simulation study using individual genetic variants as instrumental variables

			Two-stage least squares			Sequential adjustment (1)			Sequential adjustment (2)		
α_{U1}	α_{U2}	α_{U3}	β_1	β_2	β_3	β_1	β_2	β_3	β_1	β_2	β_3
0.3	0.3	0.3	0.299	0.000	-0.099	-0.039	-0.346	-0.406	-0.046	-0.432	-0.514
0.3	0.3	-0.3	0.300	0.000	-0.100	0.251	-0.199	-0.205	0.300	-0.248	-0.259
0.3	-0.3	0.3	0.300	0.000	-0.100	0.139	0.000	-0.279	0.166	0.000	-0.353
0.3	-0.3	-0.3	0.299	0.001	-0.100	0.429	0.147	-0.078	0.514	0.183	-0.099
-0.3	0.3	0.3	0.301	0.000	-0.102	0.071	-0.148	-0.080	0.085	-0.184	-0.101
-0.3	0.3	-0.3	0.301	-0.001	-0.100	0.363	0.001	0.121	0.435	0.001	0.153
-0.3	-0.3	0.3	0.301	-0.001	-0.100	0.251	0.200	0.047	0.300	0.249	0.060
-0.3	-0.3	-0.3	0.300	0.000	-0.100	0.541	0.346	0.247	0.647	0.431	0.313

Web Table 6: Mean estimates of the causal effects of X_1 (true effect $\beta_1 = 0.3$), X_2 ($\beta_2 = 0$), and X_3 ($\beta_3 = -0.1$) using three different methods from simulation study using allele scores constructed from the genetic variants as instrumental variables

Web Appendix 4

Consistency of association of genetic variants with lipid fractions and CHD risk

In order to assess the consistency of the association of genetic variants with lipid fractions and CHD risk, we constructed a lipid risk score for each variant and assessed the association of this score with CHD risk. This score was calculated using the causal estimates β_{LDL-C}, β_{HDL-C} and β_{TG} from Web Table 2 and the genetic associations X_L, X_H and X_T from Web Table 1 for each genetic variant $j = 1, \ldots, 28$ as:

$$\beta_{LDL-C}X_{Lj} + \beta_{HDL-C}X_{Hj} + \beta_{TG}X_{Tj} \tag{4}$$

The association between the lipid risk score and the log odds of CHD is plotted in Web Figure 2. If the associations of each lipid fraction are homogeneous across variants, we expect the points to lie on a straight line through the origin. With the exception of the point on the far left representing genetic variant rs2304130, this is approximately true. This variant shows an association with LDL-C and triglycerides, but no association with CHD risk and a point estimate in the opposite direction from that which would be expected based on its associations with LDL-C, HDL-C and triglycerides alone. Further investigation is needed to show whether the result for variant rs2304130, is a chance result or reflects a pleiotropic association of this variant with another risk factor.

The causal odds ratios for CHD based on 27 variants excluding rs2304130 are 0.48 (95% credible interval 0.39–0.60) per 30% reduction in LDL-C, 1.19 (95% credible interval 0.89–1.58) per 30% reduction in HDL-C, and 0.77 (95% credible interval 0.68–0.87) per 30% reduction in triglycerides.

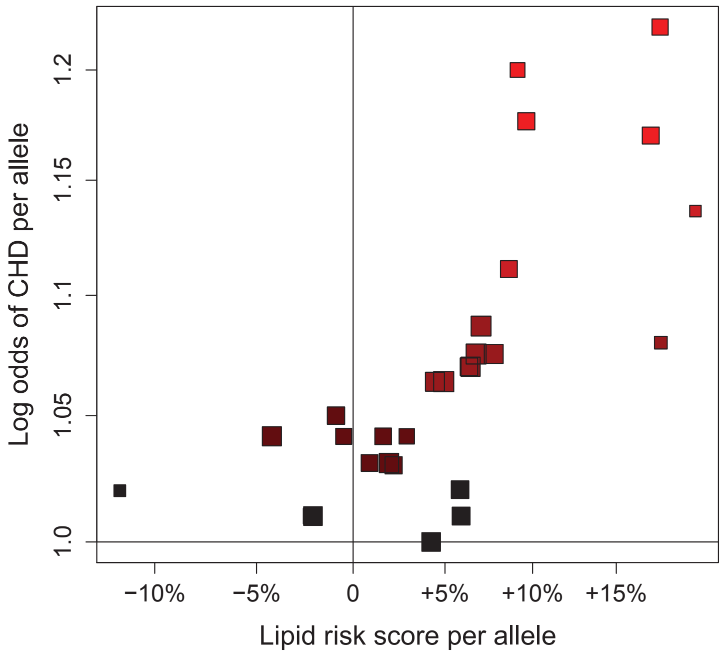

Web Figure 2: Association of coronary heart disease (CHD) risk-increasing alleles of 28 genetic variants with lipid risk score and odds of CHD (brightness and size of points: brighter points correspond to stronger associations with CHD risk, larger points correspond to more precise estimates). Note that some points are overlapping.

References

[1] Waterworth D, Ricketts S, Song K, Chen L, Zhao J, Ripatti S, et al. Genetic variants influencing circulating lipid levels and risk of coronary artery disease. *Arteriosclerosis, Thrombosis, and Vascular Biology* 2010;30(11):2264–2276.

[2] Holmes MV, Asselbergs FW, Palmer TM, Drenos F, Lanktree MB, Nelson CP, et al. Mendelian randomization of blood lipids for coronary heart disease. *European Heart Journal* 2014;[Available online ahead of print, January 27, 2014] DOI: 10.1093/eurheartj/eht571.

[3] Burgess S, Thompson S. Use of allele scores as instrumental variables for Mendelian randomization. *International Journal of Epidemiology* 2013; 42(4):1134–1144.
